# What can be learned by scanning the genome for molecular convergence in wild populations?

**DOI:** 10.1111/nyas.14177

**Published:** 2019-06-26

**Authors:** Bonnie A. Fraser, James R. Whiting

**Affiliations:** ^1^ Department of Biosciences University of Exeter Exeter United Kingdom

**Keywords:** convergent evolution, population genomics, genome scans, parallel evolution

## Abstract

Convergent evolution, where independent lineages evolve similar phenotypes in response to similar challenges, can provide valuable insight into how selection operates and the limitations it encounters. However, it has only recently become possible to explore how convergent evolution is reflected at the genomic level. The overlapping outlier approach (OOA), where genome scans of multiple independent lineages are used to find outliers that overlap and therefore identify convergently evolving loci, is becoming popular. Here, we present a quantitative analysis of 34 studies that used this approach across many sampling designs, taxa, and sampling intensities. We found that OOA studies with increased biological sampling power within replicates have increased likelihood of finding overlapping, “convergent” signals of adaptation between them. When identifying convergent loci as overlapping outliers, it is tempting to assume that any false‐positive outliers derived from individual scans will fail to overlap across replicates, but this cannot be guaranteed. We highlight how population demographics and genomic context can contribute toward both true convergence and false positives in OOA studies. We finish with an exploration of emerging methods that couple genome scans with phenotype and environmental measures, leveraging added information from genome data to more directly test hypotheses of the likelihood of convergent evolution.

## Graphical abstract

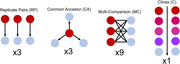

## Introduction

Convergent evolution is when independent lineages evolve similar phenotypes in response to similar selective pressures (e.g., darker fur in mice inhabiting darker soil,^1^ reduced armor in marine versus freshwater sticklebacks,^2^ repeated evolution of C_4_ photosynthesis in plants).^3^ Convergent evolution can therefore reflect both the power of selection and also its limits. Observing that similar traits have repeatedly evolved is often taken as evidence that a trait is adaptive, particularly when there is a consistent match between trait and environment. However, this phenomenon raises the question: does this pattern instead reflect a common limitation to selection and are other, better adaptive solutions possible?^4^ Examining convergent evolution, therefore, is not just interesting for its own sake, but it can help unravel the many factors known to influence adaptation, shedding light on their relative importance and the ways in which they interact. Moving through different levels of biological organization, from the phenotype through to the underlying pathways, genes, and base pairs, our predictions on what might limit selection can change (e.g., developmental constraints of pathways, limits of mutational input).^5^ Recently, with the advent of high‐throughput sequencing methods, it has become possible to explore the genomes of nonmodel organisms and ask whether phenotypic convergence is reflected at various molecular levels. This has the promise to identify loci undergoing convergent evolution and elucidate the genomic constraints and historical contingencies that lead to convergent evolution.

**Figure 1:**
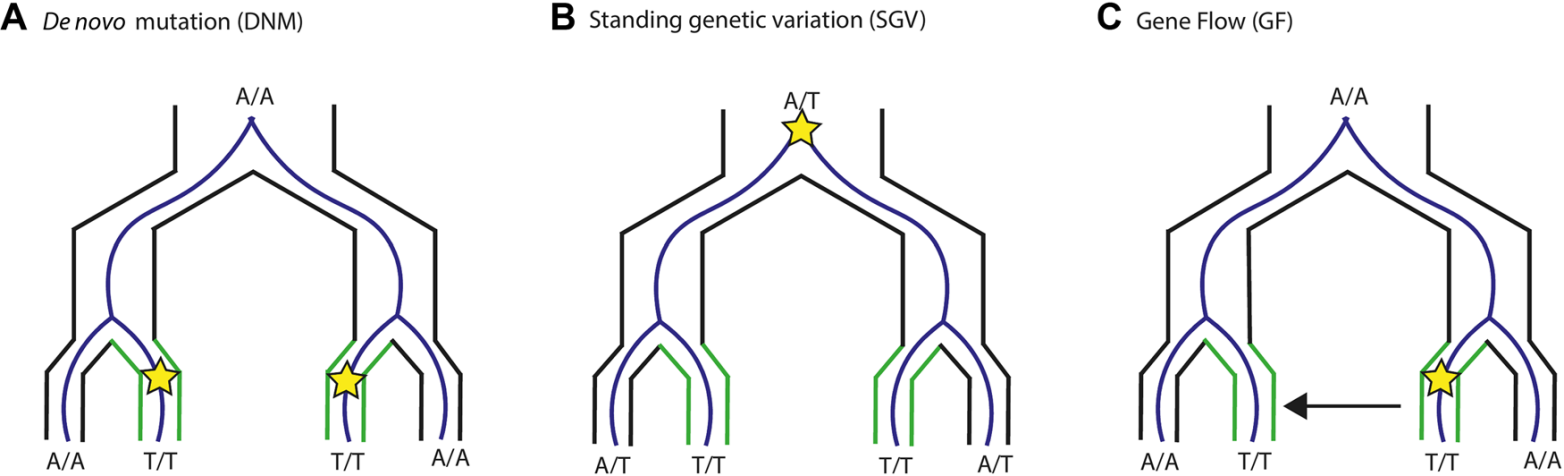
Modes of convergent molecular evolution, where independent lineages are adapting to new, similar environments (green lineages). Selection can be on (A) *de novo* mutation (mutation indicated by a star, where an A is mutated to a T), (B) standing genetic variation, and (C) gene flow (where migration between lineages, indicated by the arrow).

Molecular convergence can be achieved through a variety of modes: selection within populations can independently act on (1) *de novo* mutations (DNM) arising independently in different lineages, (2) segregating genetic variants that arose in the common ancestor (i.e., standing genetic variation, SGV), or (3) loci shared by gene flow (GF) between populations (Fig. 1). DNM convergence is often regarded as the traditional interpretation of molecular convergence because mutations are independent, but in all three modes, populations are independently adapting to similar environments.^6^ To avoid added confusion, we will adopt the framework of Arendt and Reznick^7^ and refer to all mechanisms as convergent evolution and refer to specific modes of convergence when necessary. It is worth distinguishing among these modes because each one is predicted to require different strengths of selection and reflect different limitations of evolution (e.g., selection on a DNM with low starting frequency will need to be stronger than selection on SGV with higher starting frequency to reach fixation).^6^, ^8^

The interplay between natural selection and evolutionary constraints in predicting phenotypic convergent evolution has been well explored in the literature, but we have yet to establish a framework for molecular convergent evolution. Much has been made about the role of contingency in phenotypic convergence, both the significance of random chance events, and how these can shape the context and dependency of adaptation.^9^, ^10^ Reframed in a population genetics framework, we can think of these contingencies as the importance of DNM and the processes that shape patterns of SGV. Population genetic theories of mutation–selection–drift equilibrium provide strong predictions for how DNM and SGV vary with demographic parameters (e.g., lower levels of SGV and new mutations within small versus large effective populations). However, testing these predictions in wild populations is rarely done. Similarly, the importance of constraints on phenotypic convergence (functional, developmental, and genetic) has received much consideration in the literature.^4^, ^11^ With the knowledge of entire genomes, we can begin to directly test predictions of genetic constraint.^12^ For example, we can ask whether convergently evolving loci occur more often in regions of the genome with different mutation or recombination rates. Therefore, developing a framework with clear predictions of how both population history and genomic context will affect and interact toward molecular convergence is needed and achievable.

**Figure 2:**
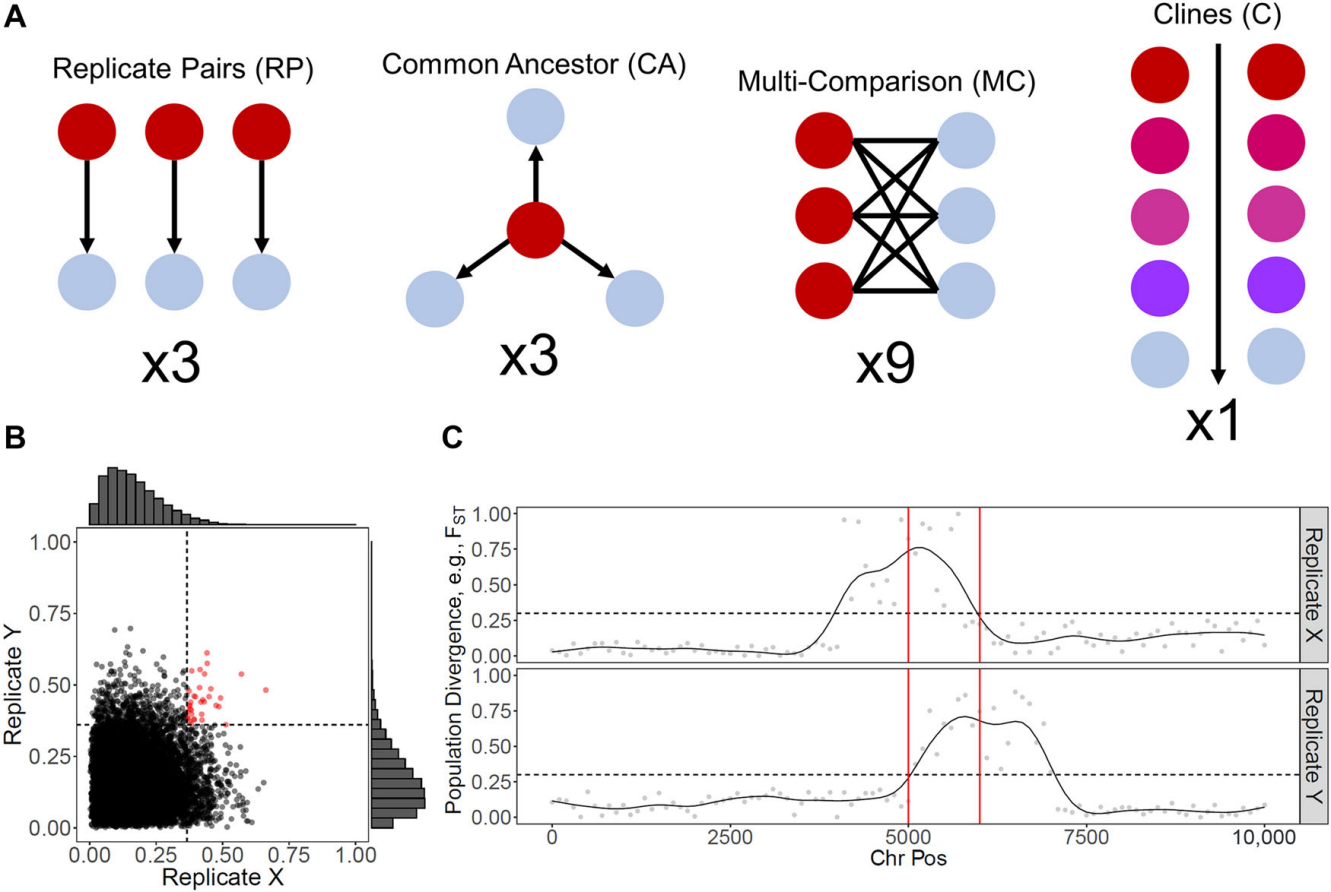
The overlapping outlier approach (OOA) used to detect convergent molecular evolution. (A) The different types of sampling design. (B) Overlapping outliers among replicates using a quantile‐based cutoff (0.95) are detected. (C) Overlapping outliers along a chromosome with an arbitrary threshold of 0.3 are visualized.

There are many different ways to detect convergent evolution at the genomic level, for example, phylogenetic comparative methods,^13^ or mapping phenotypic convergent traits using genome‐wide association studies (GWAS) or quantitative trait loci (QTLs).^14^ However, these either rely on prior knowledge of candidate genes (e.g., comparative methods) or require very large sample sizes (e.g., GWAS) or the ability to conduct controlled genetic crosses in the lab (e.g., QTL mapping). This is not useful for nonmodel species, where we have some of the most convincing and diverse evidence of phenotypic convergent evolution. Recently, a new population genomics approach has seen success in identifying convergence in the genomes of natural populations; we term this the overlapping outlier approach (OOA) (Fig. 2). This approach has the advantage of requiring little prior information about the genetic basis of adaptation, and it can be applied to a diverse range of taxa. In OOA studies, pairs of populations that have independently adapted to alternative environments are scanned at the genome level for signatures of selection, for example, population genetic differentiation/divergence. Within each set of diverging populations, selection is inferred by identifying outlying genomic areas, relative to the putatively neutral backdrop of the genome. Once outliers are identified in each replicate, the list of overlapping outliers across replicates is taken as evidence of convergent molecular evolution. Crucially, the inclusion of replicated sampling in studies of convergence differentiates the literature from studies of “local adaptation” (reviewed in Ref. 15).

Here, we review the use of OOA to studying convergent evolution in wild populations. Although experimental and domestication studies have provided valuable insights into molecular convergent evolution, they have different population and genomic structures compared with natural systems. Experimental evolution studies of convergence (reviewed in Ref. 16) have been instrumental in demonstrating the relative contributions of randomness and contingency in convergent evolution, for example, demonstrating the effects of mutations arising in a particular order on whether populations evolve convergently.^17^ These studies, however, are often limited to model species, in particular those with short generation times, and often use laboratory strains with low amounts of genetic diversity (e.g., inbred Drosophila lines or microbial lines). Consequently, the demographic parameters and genomic context are likely to be very different in these studies compared with natural populations that are outbred and highly variable. Studies of genomic convergence as a consequence of domestication have been similarly informative about molecular convergence, but again are of questionable relevance to studying adaptation in the wild. Domestication is limited to a handful of organisms and is restricted to a relatively short time frame; the strong artificial selection involved in domestication has led to inbreeding, artificially low effective population sizes, and limited diversity in comparison to wild relatives.^18^, ^19^ In addition, artificial selection for a limited number of specific traits may be quite different from natural selection acting on overall fitness in the wild, depending on how the traits relate to fitness. Therefore, investigating genomic convergence in natural populations will allow researchers to examine the effects of complex, realistic population parameters on the likelihood of convergence, while also expanding research to a diverse array of nonmodel taxa.

We begin by presenting a short synthetic review in which we analyze results from recent studies employing OOA in natural populations. We adopt a similar approach to that of Ahrens *et al*.^15^ to address and discuss how issues raised with outlier approaches in studies of local adaptation may influence OOA studies. Briefly, we examine whether features of study design and sampling intensity affect the identification of convergent loci (defined as the overlap of outliers across replicates). It is tempting to assume that false positives derived from individual outlier scans are randomly distributed across the genome and therefore will fail to repeat across replicates; however, this might not always be the case. We next explore how population demographics and genomic context can contribute toward convergence and also false conclusions in OOA studies. Finally, we explore emerging methods in the field of genomic convergence in natural populations.

## Synthetic review of OOA studies

We scanned the literature for studies that examined molecular convergence in wild systems. Searching ISI Web of Science for recent studies (2010–2018; similar cutoffs to Ref. 15 and capturing the first high‐profile studies using this method, see, e.g., Ref. 20) on convergent evolution, adaptation, and genomics resulted in a preliminary list of 441 studies (search terms are provided in the Supporting Information). From this, we manually identified and excluded studies that did not conform to the OOA approach, as well as those that employed fewer than 1000 single nucleotide polymorphisms (SNPs), that did not focus on wild systems, or that otherwise did not provide sufficiently detailed information about individual replicates. This resulted in a final data set of 34 studies. This analysis is not meant to provide an exhaustive meta‐analysis but should nevertheless be useful for highlighting trends and potential biases of OOA studies that will be informative for future studies.

Thirty‐four studies covered 24 species (Table S1, online only), consisting of fishes (N = 11 species), plants (N = 6), insects (N = 3), mammals (N = 2), birds (N = 1), and mollusks (N = 1). Between 2010 and 2018 the number of studies has increased, with more than twice as many studies occurring after 2015 (N = 23) than before (N = 11). This is presumably a consequence of the increasing availability of population genomic data sets and the field's increasing interest in questions of genomic convergence. From the resulting 34 studies, we extracted information for all pairwise comparisons of outlier analyses yielding a final data set of 238 individual OOA replicates. The degree of replication varied considerably across studies, ranging from a single comparison between two replicates^21^, ^22^, ^23^, ^24^, ^25^, ^26^, ^27^, ^28^, ^29^, ^30^, ^31^, ^32^, ^33^ to 120 comparisons between 16 replicates (all pairwise).^34^ These 120 comparisons were removed from the data set when necessary to clarify whether effects were driven by this particularly large study.

There is a difficulty in quantifying a consistent measure of “convergence” across individual studies and individual systems because, in most cases, a null expectation is difficult to define and will vary across studies.^35^, ^36^ Moreover, because the threshold of what is termed an “outlier” is variable across studies and methods, the use of qualitative similarity matrices (e.g., Jaccard's Index) across studies is impossible. For example, the expected overlap between two upper 5% quantiles is a larger proportion of the total pool than the expected overlap between two 1% quantiles. Bearing this in mind, we quantified convergence across studies as a fold‐enrichment of observed overlap against expected overlap. This null assumption simply states that there is an expected degree of overlap when comparing two subsets of a pool of data that can be calculated as the probability of achieving the same outcome given the proportion of each subset per replicate. For example, given two 5% quantiles of a total pool, we expect that 0.0025 of the total pool will occur randomly in each of the quantiles (P[A∩B] = P[A] × P[B]). This 0.0025 constitutes 5% of each of the 5% quantiles, thus the proportional overlap of the quantiles themselves is 0.05. An observed overlap of 0.1 between two 5% quantiles would therefore be a fold‐enrichment of 2 against a random expectation. We gathered information on number of outliers per replicate and total number of loci examined for each replicate, along with the observed proportion of overlapping outliers, and used it to calculate the fold‐enrichment of observed convergence relative to the simple random null. Therefore, this is not a reanalysis of original raw data presented in these studies. We explored whether enrichment was affected by various aspects of study design using a linear‐mixed modeling approach with a random effect of study to correct for multiple replicates from single studies (see Supporting Information). We found enrichment varied from 0 (no overlap)^37^, ^38^ to greater than 240,^28^ with an overall mean enrichment of 7.09 (Fig. S1, online only).

### Sampling design

We first examined the effect of choice of sampling designs common to OOA studies (Fig. 2). The most common of which was what we have termed “replicate pairs” (N_studies_ = 19; N_replicates_ = 210), and involves selecting replicated pairs of adaptively diverging populations (with the replicates known or assumed to reflect independent evolutionary lineages), performing scans within each replicate, and comparing results across replicates. This sampling design is categorized by the independence of replicates, so each pair shares an exclusive common lineage node. In contrast, “common ancestor” (N_studies_ = 1; N_replicates_ = 3) involves comparing replicated derived populations to a shared common ancestor. While still using replicated and independent “derived” populations, the comparison to a common ancestor introduces a degree of nonindependence. At the most‐extreme end of nonindependence, “multi‐comparison” study designs (N_studies_ = 4; N_replicates_ = 9) involve making multiple or even all pairwise comparisons between phenotypically divergent populations (i.e., those with phenotype *X* versus those with phenotype *Y*). Here, populations may be grouped on the basis of the presumed independent evolution of adaptations, and populations are sometimes paired in choice. For example, a design may compare a pair of populations from Europe and a pair from Asia, and then make all pairwise comparisons (Europe*_X_*–Europe*_Y_*; Europe*_X_*–Asia*_Y_*; Asia*_X_*–Asia*_Y_*; Asia*_X_*–Europe*_Y_*). Finally, “clinal” designs (N_studies_ = 10; N_replicates_ = 16), in which replicates are groups of populations examined along a gradient (e.g., latitudinal, environmental, phenotypic), are unique in taking multiple populations per replicate, as opposed to pairwise tests. Here, the methodology tends to focus on associating changing allele frequencies with the gradient in question, in what are commonly referred to as environmental association studies.

Sampling design had a significant effect on fold‐enrichment for observed convergence (LMM, likelihood‐ratio test (LRT) = 9.122, *P = *0.027), with clinal designs (log_10_‐mean ± SE = 0.976 ± 0.24) yielding approximately 10 times the enrichment observed in replicate pairs (log_10_‐mean ± SE = 0.423 ± 0.02) and multi‐comparisons (log_10_‐mean ± SE = 0.083 ± 0.173) (Fig. A). This observation was unaffected by the inclusion of the 120 replicates from Stuart *et al*.,^34^ the largest study in our data set. The effect of sampling design on enrichment could be driven by the difference in populations sampled; clinal analyses had more populations sampled per replicate (mean N_Populations_ ± SE = 33.5 ± 16.5), compared with replicate pairs (always four populations, two per replicate) or multi‐comparisons (usually three populations, with one appearing in both replicates). Indeed, Lotterhos and Whitlock^39^ found that sampling more populations increased power in outlier detection studies. Therefore, the increased enrichment in the clinal replicates could be fewer nonoverlapping false positives. However, Lotterhos and Whitlock^39^ also report that replicate pair designs outperform clinal strategies for detecting overlapping outliers under most demographic models (with the exception of island models). This analysis, however, assumes that replicate pairs are pooled together, and equal numbers of population are sampled among designs, which was generally not the case in our data set.

**Figure 3:**
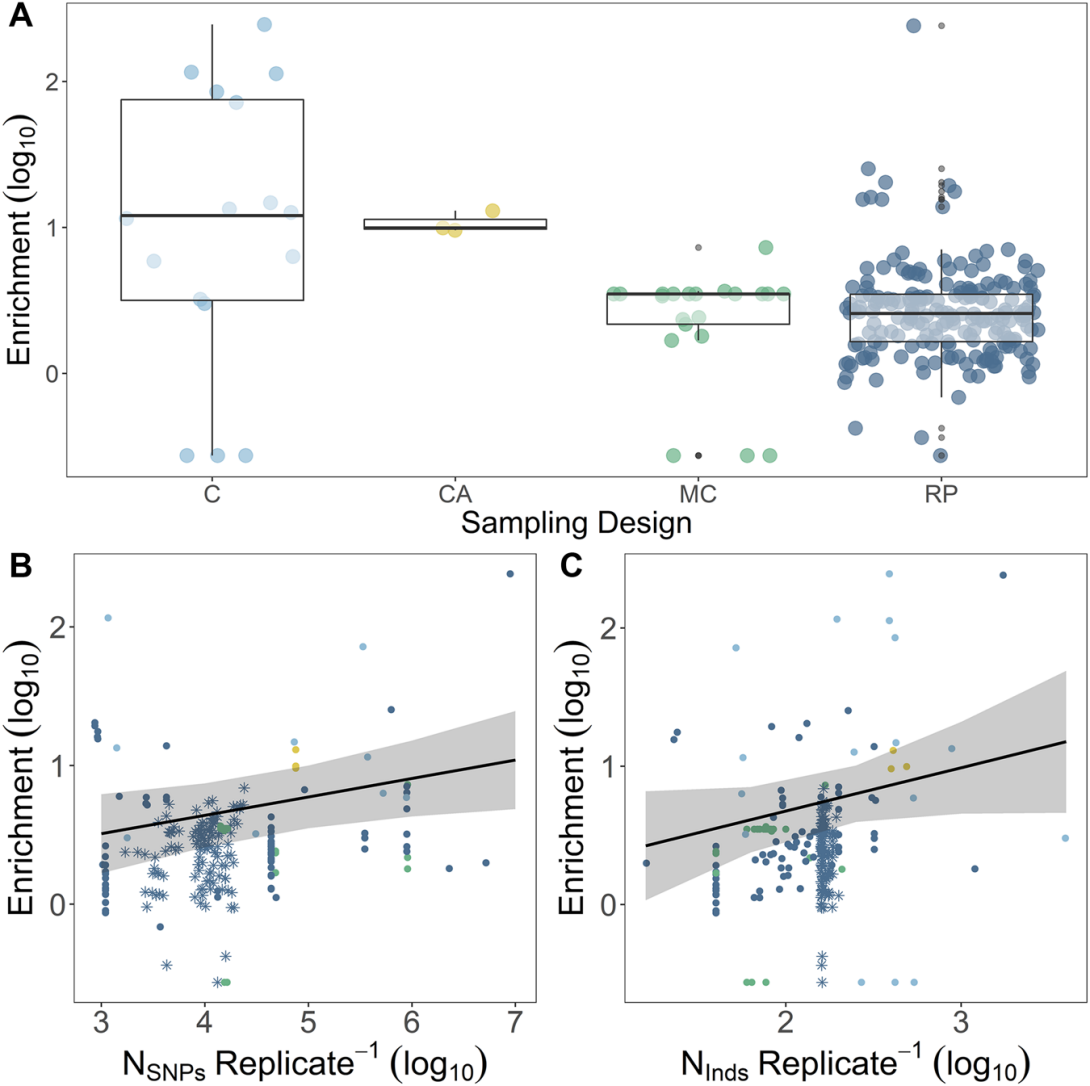
Various aspects of sampling and study design affect the amount of enrichment (i.e., detected molecular convergence). (A) Sampling design differed in reported enrichment, cline (C), common ancestor (CA), multiple comparisons (MC), and replicate pairs (RP). (B) A positive relationship was found between the number of SNPs genotyped and enrichment. (C) A positive relationship was found between the number of individuals genotyped and enrichment. In A, boxes denote 0.25, 0.5, and 0.75 quantiles, with whiskers extending to the furthest point within 1.5× the interquartile range (0.25–0.75). In B and C, the large sample of replicates from Stuart *et al*.^34^ are represented with ^*^, and lines denote model effects with 95% upper and lower confidence intervals.

### Sequencing methodology

We grouped sequencing approaches into three groups for analysis: random sequencing (i.e., nontargeted sequencing approaches such as RADseq or amplified fragment length polymorphism (AFLP)); targeted sequencing (i.e., SNPs with prior information (SNP arrays) or coding elements (RNAseq)); and, finally, whole‐genome sequencing (WGS, which mostly consisted of pooled or low‐coverage sequencing strategies in the studies reviewed). We expected targeted sequencing methods to yield high levels of enrichment because sites were chosen *a priori* to be in functional regions of the genome (i.e., exons) or sites within relevant loci. We also expected that random sequencing would show low levels of enrichment because these methods often rely on linkage between sequenced polymorphism and causative variants,^40^, ^41^, ^42^ which is likely to be variable between replicates as a product of demographic variation.^43^, ^44^ Although WGS methods also include noncoding SNPs, the exhaustive sequencing of the majority of polymorphic sites suggests that causative variants are more likely to be sequenced and are therefore available for outlier detection across replicates. This removes the dependency on linkage between sequenced SNPs and causative variants.

In testing these hypotheses, we observed that targeted methods exhibited, on average, the greatest levels of enrichment (N_replicates_ = 24, log_10_‐mean ± SE = 0.822 ± 0.10), followed by WGS (N_replicates_ = 13, log_10_‐mean ± SE = 0.640 ± 0.17), with enrichment lowest in random methodologies (N_replicates_ = 201, log_10_‐mean ± SE = 0.399 ± 0.03); however, this difference was not significant (LMM, LRT = 3.205, *P = *0.201). Similarly, Ahrens *et al*.^15^ observed no difference between targeted and random sequencing in individual studies of local adaptation. Although the general trend here agrees with our hypotheses, the effect size of sequencing methodology appears marginal and likely requires greater statistical power to evaluate fully.

We also investigated the relationship between the number of SNPs analyzed in each study and the observed enrichment. A notable and surprising trend observed by Ahrens *et al*.^15^ was that the proportion of discovered outliers was negatively associated with total SNP count in individual scans of local adaptation. We, however, observed a significant positive effect of SNP count on observed enrichment across all replicates (LMM, LRT = 5.16, *P = *0.02; Fig. B). This effect was driven by a strong correlation within the 120 replicates from Stuart *et al*.^34^ (general linear model (GLM), *F*_1,118_ *= *11.64, *P *< 0.001; Fig. S2, online only); two further analyses in which these replicates were omitted and the analysis was confined to studies that report convergent regions rather than SNPs failed to produce a significant association between SNP count and observed enrichment. The association between SNP count and enrichment in Stuart *et al*.^34^ may reflect closer ancestry between replicates with a higher number of shared SNPs and thus increased convergence if the number of shared polymorphisms is representative of coancestry.^45^ In addition, because larger SNP data sets are more likely to invalidate nonindependence assumptions between SNPs, increased observed enrichment may of course be a statistical artifact. These results should be interpreted with caution because of the potential to obtain spurious correlations between the denominator (total SNPs) and the value calculated from it (enrichment).^46^, ^47^

The subject of minor allele frequency (MAF) filtering has also received recent consideration for both its influence in detecting local adaptation signatures^15^ and influencing population structure.^48^ From a convergence perspective, the increased stringency with which rare, low‐frequency variants are removed within populations may upwardly bias estimates of enrichment in larger populations if lineage‐specific, locally adapted alleles are differentially removed during MAF filtering. Twenty‐six of our 34 studies reported MAF filters ranging from 0.01 to 0.25, although no effect was found on use of MAF filtering (LMM, LRT = 0.245, *P = *0.62) or MAF stringency (LMM, LRT = 0.0005, *P = *0.982) on observed enrichment.

An additional consideration, particularly for nonexhaustive sequencing methods, is the potential for confounding by missing data. For example, nonoverlapping outliers may represent differential coverage between replicates, such that outliers cannot overlap. If outliers cannot overlap, expectations of overlap can be adjusted accordingly, for example, by employing permutations across data sets to generate null distributions of random overlap (see, e.g., Refs. 25, 49, and 50).

### Biological sampling

OOA studies varied in both the number of individuals sampled and the number of populations sampled. We found some support for the notion that an increased number of individuals sampled per replicate is positively related to the enrichment of convergent outliers (LMM, LRT = 3.31, *P = *0.069, Fig. C). This relationship was unaffected by the inclusion of the Stuart *et al*.^34^ replicates. Similarly, in local adaptation studies, Ahrens *et al*.^15^ report a positive relationship between the number of individuals sampled and the proportion of outliers detected, supporting previous simulation work.^39^

Predictably, the number of individuals was inconsistent and potentially confounded across sequencing technologies and sampling designs, with clinal replicates including more individuals as a product of increased population sampling (N_replicates_ = 16, mean = 555 ± 233), in comparison with common ancestor (N_replicates_ = 3, mean = 433 ± 28.3), replicate pairs (N_replicates_ = 195, mean = 161 ± 10.5), and multi‐comparisons (N_replicates_ = 21, mean = 83.8 ± 9.01). By sequencing approach, individuals per replicate pair was greatest for WGS (including pool‐seq) (N_replicates_ = 10, mean = 354 ± 160) followed by targeted (N_replicates_ = 24, mean = 344 ± 161), and the lowest in random (N_replicates_ = 201, mean = 157 ± 7.54). Taken together, these observations highlight two types of study designs common to our data set: more individuals/extensive sequencing but at low coverage, and fewer individuals/random sequencing but at higher coverage, with measures of observed enrichment increasing alongside presumably, increased power per individual replicate in the former.

### Genomic sampling

Reference genomes provide the framework upon which sequenced reads are aligned, facilitating analysis of outliers within genomic regions that are typically annotated with predicted genes. We divided our studies into four well‐defined groupings on the basis of reference genome type: (1) same species (N_studies_ = 19), (2) closely related species (N_studies_ = 3), (3) *de novo* assembly (N_studies_ = 10), and (4) no reference genome, typically sequence‐level analyses across species (N_studies_ = 2). Having a reference genome of the same species is traditionally the best choice, but this option has been generally limited to model systems. For nonmodel species, the choice has typically been between using a closely related species’ reference genome or assembling reads *de novo*. A closely related species’ reference genome may provide useful information (e.g., feature annotations, chromosome‐level assembly), but these benefits can be offset by poor mapping, particularly in genomic regions that have diverged between species. The latter is of particular concern given the probable significance of these regions in studies of adaptation. In contrast, a *de novo* assembly lacks the information content and scale of a fully‐fledged reference but also avoids mapping biases. In their study of genomic convergence along parallel hybrid clines in two *Heliconius* species, Nadeau *et al*.^51^ compared results obtained through aligning *Heliconius erato* sequences to the *Heliconius melpomene* reference genome against results obtained when assembling the *H. erato* sequences *de novo*. They obtained data for ∼10× more SNPs when aligning to the related reference, which then identified an additional 56 outliers in the Ecuadorian comparison but reduced the proportion of outliers in the Peruvian comparison. Therefore, we predicted that reference genomes can have unexpected but important implications for detecting convergence.

In studies of convergence, we might expect that studies that do not use a same species‐reference to be limited in power and exhibit reduced enrichment. However, across our data set we find no significant differences in observed enrichment associated with the type of reference genome used (LMM, LRT = 4.729, *P = *0.193). Studies that do not use any reference genome display the largest enrichment (N_replicates_ = 4; log_10_‐mean ± SE = 0.942 ± 0.104), followed by *de novo* (N_replicates_ = 36; log_10_‐mean ± SE = 0.682 ± 0.11), species‐specific (N_replicates_ = 181; log_10_‐mean ± SE = 0.430 ± 0.03), and finally related‐reference (N_replicates_ = 17; log_10_‐mean ± SE = 0.124 ± 0.03).

Variable linkage is a further consideration when sampling the genome. Particularly from a convergence perspective, linkage can cause nonindependence between SNPs, violating this assumption in many outlier detection methods, and increasing the difficulty with which potentially nonindependent overlapping regions can be interpreted. In addition, variable linkage across the genome produces lower power to detect selection with reduced representation sequencing methods in regions of weaker linkage. In our data set, only 15 studies (N_replicates_ = 33) of 34 considered linkage in their analyses, with no significant effect found on observed enrichment (LMM, LRT = 2.06, *P* = 0.152).

### Analysis methods

We examined which methods were used to quantify outliers, finding F_ST_‐based methods to be the preferred choice in the vast majority of studies considered (29 of 34). Although F_ST_ was the preferred means of outlier detection, in 13 cases F_ST_‐evidence was corroborated by additional support (e.g., π, D_XY_, Tajima's D, haplotype statistics, genotype–environment associaton). F_ST_ was estimated using a variety of software, the most popular being Bayescan^52^ (N_Studies_ = 12) and Arlequin^53^ (N_Studies_ = 5). Twelve methods were used in total, including ANGSD,^54^ PoPoolation2,^55^ Hierfstat,^56^ LOSITAN,^57^ VCFtools,^58^ fdist,^59^ Stacks,^60^ and some studies calculated raw F_ST_ manually. The error rates of these various methods, incurred through often unknown parameters such as hierarchical structure and demographic assumptions of the underlying models, have been tested rigorously.^61^, ^62^, ^63^ Interestingly, Foll *et al*.^24^ examined the effect of modifying the underlying model of Bayescan to account for realistic, hierarchical structure between geographically separate replicate pairs. Their results suggest that modifying Bayescan's F‐model significantly improves detection of convergent outliers in real and simulated data compared with overlapping outliers from two separate Bayescan analyses conducted within each pair.

Although there are strong theoretical predictions of how differences in population demography will affect the ability of outlier‐scans to detect selection (see, e.g., Ref. 39), the use of methods that attempt to quantify and correct for demography is low.^15^ Several outlier detection programs now include some form of demographic correction. For example, Bayenv2^64^ includes the estimation of a matrix of covariance on the basis of neutral genetic markers, while Pcadapt^65^ incorporates a principal component analysis that accounts for hierarchical population structure. We predicted that OOA studies corrected for demography (e.g., by simulating null distributions under realistic demographic contexts, or by using a scanning method that accounts for underlying population structure and demography) will exhibit increased enrichment owing to fewer false‐positive outliers.

We divided our data set into replicates that called outliers using any means of demographic correction (N_replicates_ = 29), and those that did not (N_replicates_ = 208). We found no significant effect across the whole data set (LMM, LRT = 0.88, *P = *0.35), although enrichment was larger, on average, with correction (log_10_‐mean ± SE = 0.666 ± 0.076) than without (log_10_‐mean ± SE = 0.429 ± 0.03).

### Analysis conclusions

We have explored several features of study design that are likely to influence the detection of molecular convergence. Interestingly, sequencing aspects of study designs, such as sequencing methodology or reference genome used, exhibit minimal influence on the observed overlap of outliers. This is encouraging, particularly for nonmodel systems in which WGS or species‐specific reference genomes may be unavailable. Although, we should note that a lack of effect of sequencing design could be due to our meta‐analysis being underpowered. Sampling intensity appeared to be important and should be considered when designing future studies. We find that when the sampling power of outlier detection is limited (e.g., fewer populations sampled, or fewer individuals sampled), enrichment also decreased. This is most likely driven by an increase in false positives, which if randomly distributed within individual replicates should not overlap beyond expectations, leading to patterns of reduced molecular convergence.

## The larger context of molecular convergence

It is clear that OOA studies are on the rise, particularly those using WGS and reference genomes. But how surprised should we be that molecular convergence is reported for most studies? What does this tell us about molecular convergent evolution? In the next sections, we want to emphasize the importance of interpreting OOA studies in light of their population history and genomic context. Doing so is important not just for testing hypotheses about when and where we might expect molecular convergence to occur, but also for avoiding false conclusions about the overlap (or lack thereof) of outliers from individual genome scans. In the next sections, we first examine how different population demographic factors should influence the likelihood of each mode of molecular convergence (Fig. 1). We then explore how genomic context is predicted to influence the likelihood of convergence. Finally, we will look at how these factors can obstruct our ability to detect true convergently evolving loci in genome scans/OOA approaches.

### Importance of population demography context

**Figure 4:**
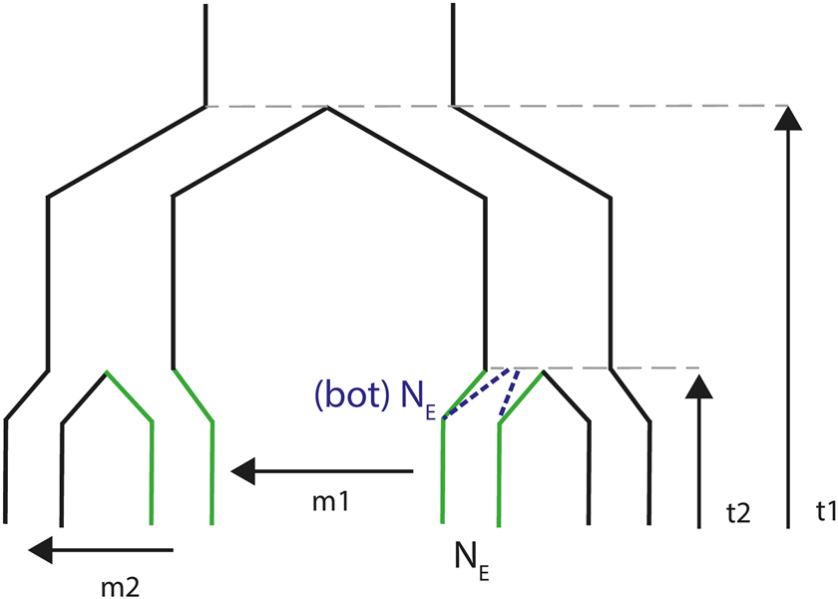
Types of demographic parameters that will affect the likelihood of convergent evolution, where independent lineages are adapting to a new similar environment (indicated in green). Parameters include founding effective populations size, for example, bottlenecks ((bot)N_E_), current effective population size (N_E_), migration between similar environments (m1), migration between different environments (m2), and time since divergence of replicates (t1) and within replicates (t2).

Accounting for variation in demography is key to predicting the likelihood of molecular convergent evolution:^66^ population demography influences the efficacy of selection and determines the variation upon which selection can act. It is therefore surprising that demography remains rarely considered in interpreting results from OOA studies. This is especially concerning given the importance of demography in detecting false positives (see below). We identify four parameters where we can make simple predictions about their effects on the different modes of genetic convergence; founding bottlenecks, current effective population sizes, migration, and time since divergence (Fig. 4).

Founding events (i.e., bottlenecks) limit the proportion of ancestral variation inherited by individual lineages in a potentially random manner by removing rare variants. Therefore, we expect strong bottlenecks to reduce the likelihood of convergence through SGV, shifting the source of adaptive variation onto DNM and GF modes (expanded on below). In a selection experiment examining the effects of bottlenecks on convergent genetic evolution in bacteria treated with antibiotics, Vogwil *et al*.^67^ observed that intermediate bottlenecks precluded genetic convergence, with increased convergence observed under strong and weak bottlenecks. Here, strong bottlenecks promoted DNM convergence through a reliance on only strongly beneficial mutations, as weakly beneficial mutations were unable to counteract the reduced fitness effects of inbreeding depression. Similarly, under weak bottlenecks clonal interference^68^ or Hill–Robertson effects^69^ appeared to lead to competition between beneficial alleles, promoting re‐use of strongly beneficial mutations and, again, increased DNM convergence.

Empirical evidence of bottleneck effects from studies of natural populations is much more indirect. Fraser *et al*.^70^ posited that low amounts of genomic convergence in naturally adapted versus experimentally introduced low‐predation Trinidadian guppies reflected differences in bottlenecking. Natural populations of guppies are typically colonized by few individuals,^70^ whereas experimental populations were founded by large numbers of guppies. Interestingly, however, Marques *et al*.^71^ observe that a large amount of ancestral standing variation is retained even in derived, phenotypically adapted stickleback populations. This retention of ancestral variation facilitated the rapid evolution of convergent phenotypes through SGV under experimental conditions.

Population size is predicted to strongly influence the efficacy of selection, with reduced size decreasing the ability of selection to remove deleterious alleles and favor advantageous alleles, leading to an increased importance of random genetic drift.^72^ With effective selection in larger populations, the likelihood of all types of convergence is increased, assuming both lineages inherit standing adaptive variation (for SGV) or experience migration from other adapted populations (for GF). Large population sizes also lead to increased mutational input, which modifies the likelihood of DNM convergence in a way mediated by both the complexity of the fitness landscape^73^, ^74^ and the degree of redundancy in mapping genotype to phenotype (i.e., the number of genetic routes to the same phenotype, GP‐redundancy).^35^ For example, under a simple fitness landscape with a single adaptive peak and low GP‐redundancy, increased mutational input will promote a more rapid DNM convergence. On the same simple fitness landscape with high GP‐redundancy, increased mutational input will promote phenotypic convergence, but through potentially divergent genetic routes in independent populations. Such a process, however, may be constrained by Hill–Robertson effects if multiple genetic routes of contrasting fitness occur within the same asexual population. Indeed, the significance of Hill–Robertson interference has been invoked to explain patterns of increased DNM convergence with varying population sizes across microbial experimental studies.^75^ Finally, on a complex, rugged landscape with multiple fitness peaks, increased mutational input is expected to drive divergent outcomes.^76^ Divergence may occur through both the ascension of different peaks on a rugged landscape, and also through very large populations possessing the ability to cross fitness valleys, as polymorphic individuals exist within the fitness valley long enough for escape genotypes to emerge.^74^

GF is also highly variable across natural systems and is predicted to affect convergence. Here, we discuss its implications for genomic convergence in two forms: GF between populations experiencing similar environments (m1; Fig. 4) and GF between diverging populations (m2; Fig. 4). In the first of these scenarios, GF facilitates adaptive allele sharing (required for the GF model of convergent evolution) and increases overall variation. The sharing of adaptive alleles may be particularly important for natural populations, in which a reliance on adaptive alleles arising *de novo* may constrain rapid adaptation and increase extinction risk.^77^, ^78^ GF between diverging populations, if excessive, can constrain local adaptation and decrease the likelihood of convergence across replicates. Moderate GF, however, increases the efficacy of selection around locally adapted alleles and limits the degree of random drift at neutral sites,^79^, ^80^ which for OOA studies is expected to improve outlier detection, and thus improve inference on those outliers that overlap across replicates.^81^ In one of the few studies to consider the influence of demography on genomic convergence, Rougemont *et al*.^82^ detected fewer numbers of outliers in sympatric, introgressing replicate species pairs of lampreys (*Lampetra*) compared with isolated parapatric pairs. The outliers found in introgressing replicates, however, exhibited much stronger signals of molecular convergence when compared with one another, potentially as a reduction in false positives through improved outlier detection.

GF also has the potential to mediate clustering of adaptive alleles within genomic regions that are intrinsically resistant to introgression and recombination. For example, in the presence of divergent selection, locally adapted haplotypes that are harbored within recombination‐suppressing inversions are predicted to be maintained even in the face of GF;^83^ but see Ref. 84. This mechanism is therefore expected to promote convergence if chromosomal rearrangements are common across lineages. Recent studies of *Littorina saxatilis* crab‐wave ecotypes, in particular, have highlighted the potential significance of recombination‐suppressing inversions in producing genomic convergence.^85^, ^86^, ^87^

Finally, it is intuitive to predict that the timescale of divergence will affect the likelihood of convergence through SGV, as shared variation will decrease, and *de novo* variation will increase with time since a common ancestor (t1 in Fig. 4). This has been demonstrated by Conte *et al*.^45^ where, in a meta‐analysis of molecular convergence, the signature of convergence was negatively correlated with lineage age. Nevertheless, the time dependency of shared versus unique genetic variation will depend on other demographic parameters, with parameters such as population size and mutational input modifying the rates at which independently derived variation is acquired and shared variation is lost.

### Importance of genomic context

Similar to demography, genomic factors, such as mutation and recombination rate, will affect diversity and the efficacy of selection across the genome. Heterogeneity of the genomic landscape is a double‐edged sword for studies of convergence in wild populations. On the one side, there are well‐supported predictions for the roles of mutation and recombination rates in the adaptation process. On the other side, heterogeneity in mutation and recombination can influence nonadaptive processes and, in some cases, resemble patterns of selection, confounding genome scan analyses. Consequently, careful evaluation of genomic context is important in OOA studies of convergent evolution. This may be difficult in nonmodel species without a reference genome, recombination map, or estimates of mutation rates. However, factors such as surrounding diversity and linkage disequilibrium can be reported from most sequencing strategies and allow us to put the outliers into their genomic context.

Mutation is the substrate of evolution, and it follows that variation in mutation rate across the genome will correlate with variation in molecular convergent evolution. Orr^88^ found that the probability of convergent evolution occurring at a given gene is simply a function of the number of beneficial mutations that are possible at the gene. Increased mutation rate has been invoked to explain patterns of repeated adaptation in cases such as the *Pitx1* gene in three‐spined stickleback.^89^ *Pitx1* is involved in the repeated evolution of pelvic loss in freshwater habitats and exhibits an elevated mutation rate that stems from an increased thymine–guanine content, which leads to a subsequent increase in double‐stranded DNA breakage.^90^ There are other genomic features that correlate with variation (and thus potentially mutation rate) (reviewed in mammals in Ref. 91), including distance from telomeres, GC‐content, repeat content,^92^, ^93^ and recombination rate. The nonrandom nature of these processes across lineages should influence the likelihood of DNM convergence, as well as clustering variation within the genome that may subsequently evolve convergently through SGV modes.

Depending on the local genomic context of a beneficial locus, both increased and decreased recombination rates can increase the chances of molecular convergence. Reduced recombination can favor the maintenance of beneficial haplotypes; it appears to have contributed to the convergence of social chromosomes across ant species,^94^ and reduced recombination is a feature of recurrent genomic regions linked to repeated adaptation in three‐spined stickleback^95^ and parallel speciation in cichlids.^29^ However, high recombination also reduces hitchhiking, and can therefore decouple advantageous alleles from disadvantageous ones and increase the efficacy of subsequent selection. This reduction in linked positive selection and background selection (BGS)^96^, ^97^ in areas of high recombination is predicted to drive positive correlations between recombination rate and nucleotide diversity genome‐wide. Indeed, a positive relationship between recombination and nucleotide diversity has been observed in several species, including chimpanzees^98^ and *Drosophila*,^99^, ^100^ but its ubiquity among taxa is questionable.^101^, ^102^

Given that these features of the genomic landscape are important for promoting or limiting adaptation, if they are consistent across lineages, then we would predict some degree of convergence at the genome level. Landscapes of nucleotide diversity in birds have been shown to correlate across populations of warbler^103^ and even across species.^104^, ^105^ However, it is less clear whether the mutational and recombination processes that shape these diversity patterns are likewise stable among populations and across species boundaries. Mutation rates are only now being directly measured from WGS of parent–offspring trios (see, e.g., Ref. 106) and it therefore remains to be seen how broadly mutation rate is conserved. Recombination intervals have also been recorded as conserved across species,^107^ and a conserved genetic basis of recombination rate variation has been documented across mammals.^108^ However, recombination landscape divergence has also been recorded,^109^ and the capacity for recombination rate to vary across individuals and evolve may hinder its fine‐scale conservation across lineages. Importantly, broadly conserved genomic landscapes can also confound genome scans, where repeated patterns of reduced diversity caused by nonadaptive processes may be misinterpreted as evidence for selection (further discussed below).

Most OOA studies have focused on SNPs, but structural variants (SVs) are predicted to underlie adaptation as well. SVs constitute several types of sequence variation: copy number variants (CNVs) represent variation in sequence duplication, inversions occur when sequence orientation is reversed, and insertions and deletions (indels) denote the gain and loss of sequence, respectively. CNVs can have a mutation rate of 100–1000 times greater than SNPs.^110^, ^111^ Indeed, SVs may be the largest source of standing variation across the genome. For example, a recent evaluation of standing structural variation in a wild, nonmodel fish (*Chrysophrys auratus*) found thrice the levels of structural variation in comparison to SNP variation.^112^ If certain genes or gene families are both prone to CNV and ecologically adaptive, we expect a higher probability of molecular convergence in these genes. For example, β‐defensins that are important for innate immunity are enriched for CNVs and have undergone convergent evolution in humans and macaques,^113^ parallel clines of CNV allele differentiation have been recorded in North American and Australian *Drosophila melanogaster*,^114^ and convergent evolution of CNVs has been recorded in marine–freshwater stickleback.^115^ Unfortunately, genome scans are poorly equipped to deal with these forms of molecular genetic data—especially studies without access to a reference genome. However, the advent of long‐range sequencing technologies has improved the detection of SVs and therefore testing whether they are undergoing convergent evolution across lineages should now be in reach for nonmodel systems.

### False positives in genome scans

Although selection occurs on variation in a population, the signature of selection in population genomic studies is often a reduction in diversity, that is, where a selective sweep has fixed an adaptive locus and its linked variation. False positives can arise if regions of the genome have reduced diversity for reasons other than positive selection. Specifically, BGS, where selection against deleterious alleles can cause a reduction in genetic variation at linked neutral sites,^96^ is predicted to be variable across the genome because of heterogeneity in recombination and mutation, which means that false‐positive signatures of selection may also be heterogeneous across the genome. The intensity of BGS will depend on population demography; for example, the associations between population size and linkage between polymorphic sites are predicted to mediate the effects of BGS around the genome. BGS has been observed to increase in contracting populations, including humans^116^ and *Drosophila*,^117^ although theoretical predictions suggest BGS may be strongest in small‐intermediately sized populations.^118^

It is tempting to assume that the replication design inherent to OOA studies will ensure that false‐positive signals of convergence are rarely encountered, but if genomic landscapes are evolutionarily stable, OOA studies may be biased toward the identification of similar false‐positive outliers in independent lineages. For example, we have discussed how both recombination and genetic diversity tend to be generally stable across broad taxa (see caveats detailed above).^104^, ^119^, ^120^ We expect, therefore, some degree of outlier overlap because of shared genomic landscapes rather than shared responses to selection. The significance of linked selection in producing heterogeneous landscapes of genomic differentiation has been invoked for *Ficedula* flycatchers,^121^ and the emergence of common differentiation landscapes over time through linked selection has been observed in adaptive radiations of monkeyflowers.^122^ Recent simulation work has also confirmed the above, demonstrating that overlapping F_ST_ outliers frequently occur in replicate population pairs with ineffective natural selection.^81^

There has been recent debate over the relative susceptibility of population differentiation statistics used in selection scans (i.e., F_ST_ and D_XY_) to BGS. F_ST_ is a relative measure and is therefore dependent not just on how populations have diverged, but also on the amount of variation within each population: F_ST_ outliers can indicate among‐population allele frequency differentiation or reduced variance within populations. By contrast, D_XY_ 123 is an absolute measure of divergence, driven predominantly by sequence variation rather than allele frequencies. Cruickshank and Hahn^124^ argued that because BGS decreases within‐population diversity, F_ST_ outliers are more susceptible to BGS than D_XY_ (also see Ref. 125). However, by investigating simulated populations under varying demographic scenarios, Matthey‐Doret and Whitlock^126^ found that BGS influenced global diversity as well as within population diversity, and that D_XY_, not F_ST_, was affected by BGS. Importantly, this nullifying effect of BGS occurred only with GF between the diverging populations.

Given the confusion over this debate, it is now becoming common to look at the overlap of multiple parameters when testing for selection. This was the case for 13 studies in our data set, in which another measure (e.g., D_XY_, π) was used to corroborate F_ST_ outlier loci. However, we would like to emphasize caution with this approach, as the different measures themselves can be correlated for reasons other than selection, making it inappropriate to simply assume that outliers shared across different metrics are strong evidence of selection. F_ST_ and D_XY_, for example, can be positively^127^ or negatively^103^, ^105^, ^122^ correlated depending on the respective presence or absence of GF. Although these measures are generally well correlated regardless of demography in very young lineages, the correlation breaks down with increased divergence in allopatry.^81^ Investigations of simulated populations under different demographic parameters have observed that shared F_ST_ and D_XY_ outliers depended on demography and the efficacy of selection, where overlaps between the measures occurred in either (1) simulated genes under strong selection under demographies with effective selection, or, (2) as for overlapping F_ST_ outliers, simulated genes with common gene features, such as diversity and coding proportion under demographies with ineffective selection.^81^

We argue that, rather than combining potentially inconsistent measures, the best approach is to estimate past demography and choose the appropriate outlier detection method accordingly. Importantly, quantifying past demography could provide insights into the demographic conditions most conducive to convergent molecular evolution, either in a study with multiple contrasts or in a meta‐analysis such as this one. There are now multiple analysis options for estimating demography on the basis of features of the allele frequency spectrum (e.g., ∂a∂i,^128^ fastsimcoal2,^129^ ABC approach^130^). Distinguishing the effects of different demographic parameters on convergent evolution in the wild will be difficult but offers a fruitful avenue of future research given its current underappreciation in studies of convergence.

## Outlook

The likelihood of molecular convergence will depend on many factors beyond population history and genomic context, but these questions are not easily answered by genome scans alone. Genome scans cannot identify causative loci; in the case of WGS, outlier windows often contain many possible candidate genes and in the case of reduced representation sequencing, outliers rely on strong linkage to the unknown, causative loci. Furthermore, the phenotype‐to‐genotype map, the multivariate similarity of the environment, and the pathway of molecular convergence (Fig. 1) will all influence the likelihood of molecular convergence. The study of convergence therefore requires approaches that address these issues directly; it cannot simply be an extension of local adaptation genome scan studies. New methods are emerging that couple genome scans with phenotypic and environmental data and that leverage added information from genome data, such as co‐ancestry of alleles and gene function data to more directly test hypotheses of the likelihood of convergence. In this last section, we will briefly highlight some of these methods that we believe will help the field develop a more complete view of molecular convergence.

### Genetic constraints and their effects on the phenotype

Genetic constraints have long been recognized as factors that will affect convergent evolution since they determine which evolutionary paths are accessible. Because they are less likely to have negative side effects on fitness, genetic changes that affect fewer epistatic interactions (gene–gene interactions) or that have fewer pleiotropic effects (genes affecting multiple phenotypic traits) are predicted to more frequently be involved in adaptive evolution.^131^, ^132^ However, increased pleiotropy and epistasis may increase the chances of convergent evolution because they can restrict evolution to a limited number of genotype–phenotype paths. The influence of epistasis on molecular convergence should depend on whether replicated phenotypes are derived from a common ancestral state, and by extension share a common genetic background. With a common genetic background, epistasis is predicted to constrain evolution to use similar genetic architectures; whereas on divergent genetic backgrounds, and presumably different epistatic interactions, epistasis works against molecular convergence and selection may favor divergent outcomes.^133^ Clearly, how the genotype maps to the phenotype, and how much redundancy exists within these maps, will have implications for convergent evolution.^35^ Genome scans link the genome to selection, but they cannot inform us of the genotype–phenotype map; doing so requires connections between population genomics and quantitative and functional genetics.^134^

That said, the question of genetic constraint has been indirectly assessed with genome scans through a variety of related analyses. For example, it has been argued that cis‐regulatory regions are more likely to underlie adaptive evolution, being subject to lower levels of negative pleiotropy and encompassing a larger mutational target than coding regions.^135^ Jones *et al*.^136^ found that the majority of SNPs associated with convergent marine to freshwater adaptation in sticklebacks were found in noncoding regions. Epistasis may also affect whether outliers are detected. By simulating QTLs under selection, Jones *et al*.^137^ found that epistasis reduces mean F_ST_ for causative QTL by spreading the signal of selection among interacting loci, making them more difficult to detect in outlier scans. Finally, to more directly test the effects of constraints, Yeaman *et al*.^35^ presented an analysis to estimate the number of loci that could potentially contribute to an adaptation and an index to quantify the total number of constraints that contribute to repeatability, relative to the null hypothesis of no constraints.

Outlier approaches may also be biased for genes of large effect, while quantitative traits are predicted to be due to many small effect loci.^138^ However, it may be the case that molecular convergence is more likely to involve large effect loci. Through modeling molecular convergence, MacPherson and Nuismer^139^ found that SGV convergence is more likely on single large effect loci, simply because the probability of fixing one locus in multiple independent populations is higher than fixing multiple loci many times. As selection efficacy decreases (e.g., through smaller starting allele frequencies or smaller population sizes), the probability of fixing more than one locus also decreases. Tools are now being developed that tackle this question directly by looking at genetic convergence at a pathway rather than loci level. The methods developed by Daub *et al*.^140^, ^141^ take advantage of functional pathway information before conducting outlier scans. This method has the potential to detect small polygenic signals not found in traditional outlier approaches. More generally, there is a growing interest in detecting polygenic signals of adaptation using population genomics data,^142^, ^143^, ^144^ with emerging machine‐learning methods such as Random Forests offering a tractable alternative for analyzing existing SNP data sets (see, e.g., Refs. 145 and 146). A better appreciation of genotype–phenotype redundancy will be essential for answering questions in convergent polygenic adaptation.

### Importance of environmental context

The implicit assumption in overlapping outlier tests is that the selection environment is common among replicates, both with reference to the specific optima favored by selection and in the power with which selection acts. Indeed, 10 of the studies reviewed here looked for molecular convergence among “ecotypes,” that is, populations adapted to diverging environments through a suite of phenotypic traits. However, natural environments are highly dimensional^147^ and any two environments that share a common feature (e.g., predation) may be highly divergent in a range of other selective agents (e.g., parasitism) that may preclude convergence if the relevant traits are linked at either a genetic or fitness level.

There are multiple approaches available to researchers that correlate features of the environment to SNP frequencies (e.g., Bayenv2,^64^ Pcadapt,^65^ and latent factor mixed models (LFMM)^148^) Here, genome scans will need to be coupled with careful sampling of relevant environmental parameters, which may need to be scaled down to reduced dimensionality through multivariate approaches. Examining multiple pairs of populations distributed along a continuum of environmental gradients rather than discrete replicated environments can open up many other systems and questions, for example, the role of the environment in the evolution of nonparallel traits.^149^ Recent work has also begun to explore how much convergence is expected given the similarity of environment, and indeed whether environmental similarity predicts genomic convergence from a geometric perspective.^150^ Stuart *et al*.^34^ employed a vector approach that quantified multidimensional vectors for phenotypic, environmental, and genetic (neutral markers) variation across 16 replicated lake‐stream stickleback ecotypes. By examining all pairwise comparisons of each lake‐stream replicate, this study was able to explicitly test whether angles (representing the direction of vectors through multidimensional space) and differences in length (representing the similarity in the amount of variation within each vector) of phenotypic and environmental vectors could predict outlier sharing. Their findings confirmed that outlier sharing was positively associated with replicates that were environmentally or phenotypically more similar. Thompson *et al*.^151^ employed a similar multivariate vector approach to examine how dissimilarity of environments may limit molecular convergence via SGV in simulations. Their results suggest that even small deviations of selection within complex, multidimensional space may limit SGV convergence.

### Modes of molecular convergence

We started out by detailing the different modes of convergent evolution (DNM, SGV, GF; Fig. 1) and explored their likelihood under different scenarios. Until recently, different modes of convergence were either explored separately or ignored in the study of molecular convergence. GF convergence has been primarily investigated by looking for signatures of introgression on the basis of an *a priori* assumption that GF was the most likely pathway to convergence.^152^ Additionally, genome‐scan approaches, concerned with detecting sweep signatures, will be biased toward stronger sweeps on DNM, rather than potentially weaker sweeps on standing or introgressed variants.^153^ Therefore, more sophisticated methods to detect SGV are needed. Selection on DNM and SGV is predicted to leave different signatures in variation on linked neutral sites.^154^ Roesti *et al*.^155^ predict SGV convergence to leave a “peak‐valley‐peak” signature of genetic differentiation (i.e., a region of low genetic differentiation bordered by regions of high levels of differentiation), and find supporting evidence in sticklebacks when comparing F_ST_ of freshwater–marine comparisons to freshwater–freshwater comparisons. This signature reflects the expected low differentiation around the shared causative loci and high differentiation of lineage‐specific hitchhiking regions at either flank.

Recently, Lee and Coop^6^, ^36^ developed a framework that distinguishes among modes of convergent evolution. This framework is based on examining the coancestry patterns of selected haplotypes, with each mode of convergence exhibiting a unique haplotype phylogeny and subsequent signature of coancestry (fig. 2 in Ref. 6). Briefly, coancestry coefficients are generally high, particularly around the selected allele, under GF modes; generally low across the haplotype, but peaked around the selected allele under SGV; and consistently low across DNM haplotypes. Measuring coancestry coefficients both within and among populations experiencing similar selection can then be used to calculate composite likelihoods scores of observed allele frequencies given a specific mode of convergence.

These methods are locus‐specific tests, but many studies report several convergent loci, suggesting the potential for a combination of modes of molecular convergence to underlie convergent adaptation. Indeed, Pease *et al*.^156^ employed a phylogenomics approach to demonstrate that adaptive variation in a radiation of a wild tomato clade can be traced to shared ancestral standing variation, introgression between lineages, and common DNM across lineages.

## Conclusions

Research using the OOA in natural populations can provide valuable insight not only into the repeatability of evolution but also evolution's limitations and constraints. The accessibility of this approach means diverse systems can be studied at an increasingly larger genome scale. We are therefore beginning to test hypotheses concerning where and when we might expect molecular convergence in real‐world systems. Unfortunately, this approach can also be prone to false positives. Our review found that studies that maximize biological sampling power within replicates will be better placed to avoid false positives and therefore identify loci responsible for convergence. However, demographic and genomic context can mislead OOA studies; crucially the effect of these issues on outlier tests can be nonrandom, potentially leading to overlap among independent replicates. Furthermore, demographic variation and genome landscapes need to be considered when investigating the likelihood of convergence, and their variation among natural populations will provide fruitful systems for exploring the nature of molecular convergence. Finally, there is now a movement toward a unifying framework for connecting population genomic analyses to molecular convergence that is helping the field grow beyond its origins as a simple extension of local adaptation studies, making this an exciting time to be studying molecular convergence.

## Competing interests

The authors declare no competing interests.

## Supporting information


**Figure S1**. Histogram of log_10_‐transformed enrichment values across all 238 replicates included in the analysis. The dashed red line denotes the mean.

**Figure S2**. Positive association between enrichment of overlapping outliers and SNP count across the 120 comparisons within Stuart *et al*.^34^

**Table S1**. Systems and references explored through OOA studies used in our synthetic review
