# Supplementary figures and images for: What can be learned by scanning the genome for molecular convergence in wild populations?
Source: Ann N Y Acad Sci. 2019 Jun 26;1476(1):23–42. doi: 10.1111/nyas.14177 (PMC7586825; doi:10.1111/nyas.14177)

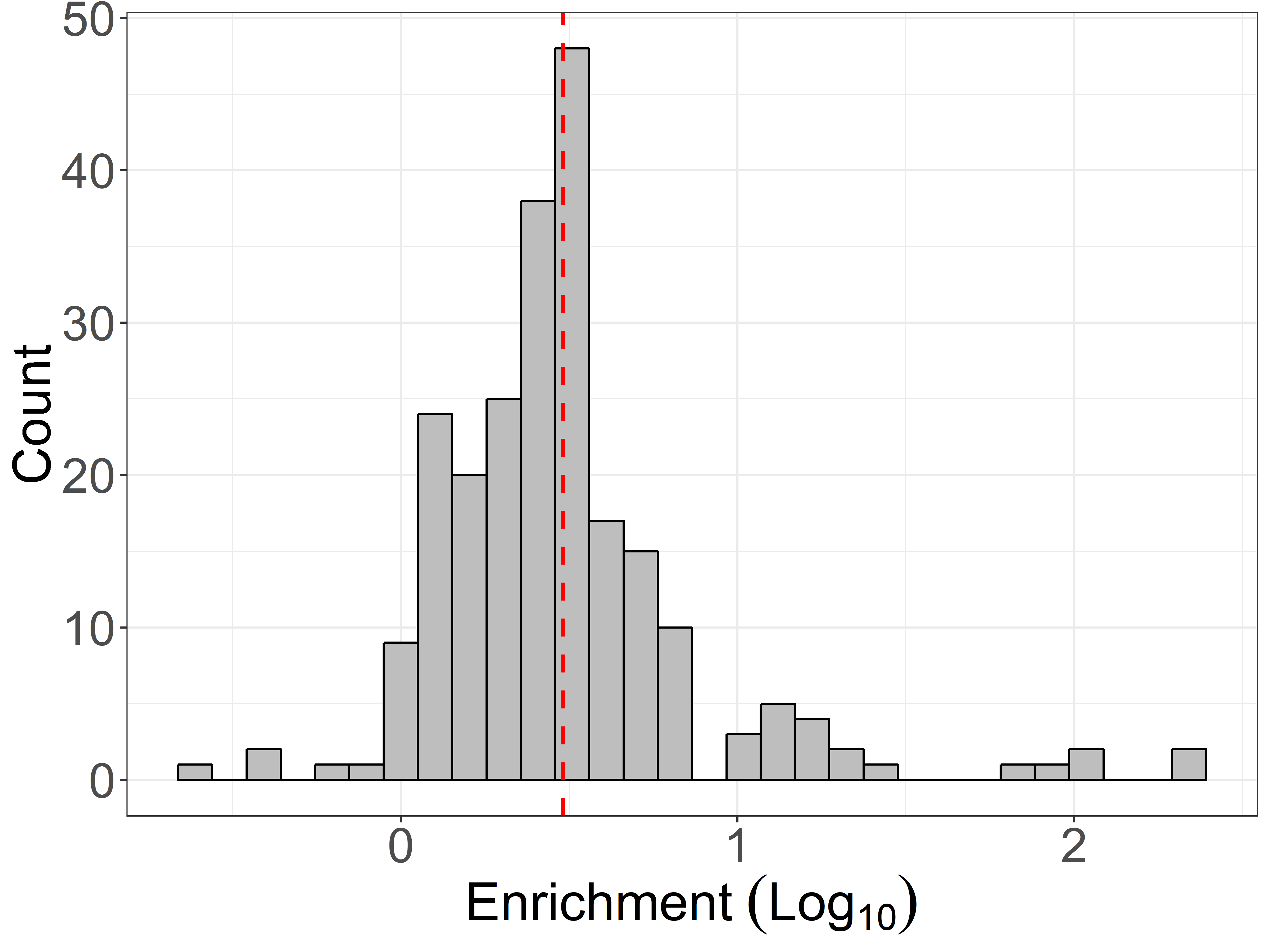

Supplement: Supplementary file 1 — Figure S1. Histogram of log10‐transformed enrichment values across all 238 replicates included in the analysis. The dashed red line denotes the mean. [file NYAS-1476-23-s001.tif]

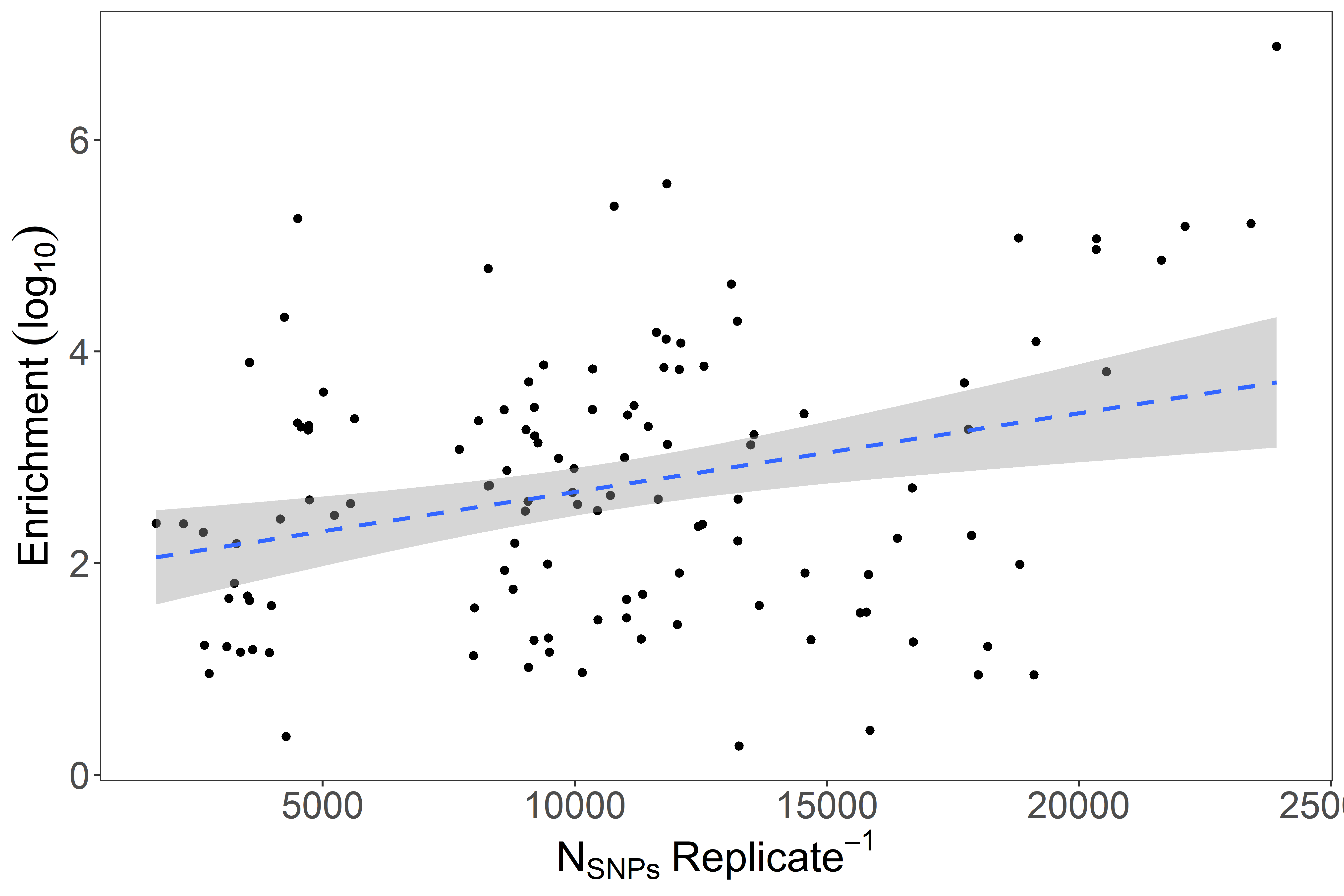

Supplement: Supplementary file 2 — Figure S2. Positive association between enrichment of overlapping outliers and SNP count across the 120 comparisons within Stuart et al.34 [file NYAS-1476-23-s002.tif]
