# Supplementary material for: What can be learned by scanning the genome for molecular convergence in wild populations?
Source: Ann N Y Acad Sci. 2019 Jun 26;1476(1):23–42. doi: 10.1111/nyas.14177 (PMC7586825; doi:10.1111/nyas.14177)
Supplement: Supplementary file 3 — Table S1. Systems and references explored through OOA studies used in our synthetic review [file NYAS-1476-23-s003.docx]

| **Table S1**: Systems explored through OOA studies incorporated in our synthetic review. Asterisks denote studies for which separate analyses were performed over different datasets in the same study (handled as separate studies in analyses). | | |  |
| --- | --- | --- | --- |
| **Study system** | **Taxa** | **Convergence** | **References** |
| Old and New World Vultures | Bird | Obligate scavenging lifestyle | Ref. [1](https://paperpile.com/c/ymiB3v/u8goY) |
| Three-spined stickleback (*Gasterosteus aculeatus*) | Fish | Marine - Freshwater Ecotypes (physiology, armour plating) | Refs. [2,3](https://paperpile.com/c/ymiB3v/AjK7Y+aef1H) * |
|  |  | Lake - Stream Ecotypes (shape, trophic morphology) | Refs. [3–5](https://paperpile.com/c/ymiB3v/OimWD+hIHO5+aef1H) |
| Atlantic Salmon (*Salmo salar*) | Fish | Latitudinal clinal adaptation | Ref. [6](https://paperpile.com/c/ymiB3v/0Mb7f) |
|  |  | Anadromous - Freshwater Ecotypes | Ref. [7](https://paperpile.com/c/ymiB3v/mKlti) |
| Lampreys (*Lampetra fluviatilis/planeri*) | Fish | Parasitic - Non-parasitic ecotypes | Ref. [8](https://paperpile.com/c/ymiB3v/yA8Lu) |
| Lake Victoria cichlids (*Pundamilia sp*) | Fish | Replicated species pair | Ref. [9](https://paperpile.com/c/ymiB3v/PlxXs) |
| Nicaraguan crater lake cichlids (*Amphilophus citrinellus*) | Fish | Parallel crater radiations | Ref. [10](https://paperpile.com/c/ymiB3v/Jigx5) |
| Killifish (*Fundulus heteroclitus*) | Fish | Pollution-tolerance | Ref. [11](https://paperpile.com/c/ymiB3v/AoKJG) |
| Killifish (*Lucania parva/goodei*) | Fish | Osmotic-tolerance | Ref. [12](https://paperpile.com/c/ymiB3v/r39qY) |
| Atlantic Herring (*Clupea harengus*) | Fish | Annual spawning season | Ref. [13](https://paperpile.com/c/ymiB3v/mjJTK) |
| Atlantic Cod (*Gadus morhua*) | Fish | Adaptation to temperature | Ref. [14](https://paperpile.com/c/ymiB3v/wll5y) |
| Molly (*Poecilia mexicana*) | Fish | Hydrogen Sulphide-tolerance | Ref. [15](https://paperpile.com/c/ymiB3v/fISYS) |
| Guppy (*Poecilia reticulata*) | Fish | High - low predation ecotypes | Ref. [16](https://paperpile.com/c/ymiB3v/DZIU8) |
| *Drosophila melanogaster/hydei* | Insect | Latitudinal clinal adaptation | Ref. [17](https://paperpile.com/c/ymiB3v/9sIdU) * |
| *Heliconius melpomene/erato* | Insect | Wing colouration (along hybrid clines) | Ref. [18](https://paperpile.com/c/ymiB3v/JNhxX) |
| Humans (*Homo sapiens*) | Mammal | Skin colour | Ref. [19](https://paperpile.com/c/ymiB3v/b8aNQ) |
|  |  | Altitudinal adaptation | Ref. [20](https://paperpile.com/c/ymiB3v/mvA5E) |
| Pika (*Ochotona princeps*) | Mammal | Adaptation along elevational gradients | Ref. [21](https://paperpile.com/c/ymiB3v/ST2tq) |
| Periwinkle (*Littorina saxatilis*) | Mollusc | Crab - Wave ecotypes | Ref. [22–24](https://paperpile.com/c/ymiB3v/yK0QX+hKlpW+PGrR8) |
| *Arabidopsis halleri* | Plant | Adaptation to altitude | Ref. [25](https://paperpile.com/c/ymiB3v/qCQ51) |
| *Heliosperma pusillum* | Plant | Alpine - Montane Ecotypes (leaf, indumentum, trichome morphology) | Ref. [26](https://paperpile.com/c/ymiB3v/9EvTg) |
| Weedy rice (*Oryza spp.*) | Plant | Agricultural weed syndrome' | Ref. [27](https://paperpile.com/c/ymiB3v/j4gey) |
| Cottonwood (*Populus deltoides/trichocarpa*) | Plant | Adaptation to local climate | Ref. [28](https://paperpile.com/c/ymiB3v/UKqLg) |
| Lodgepole Pine (*Pinus contorta*) / Interior Spruce (*Picea spp.*) | Plant | Adaptation to climate | Ref. [29](https://paperpile.com/c/ymiB3v/O8xMQ) |
| Eucalypts (*Eucalyptus tricarpa/locophleba/salubris*) | Plant | Adaptation to aridity | Ref. [30](https://paperpile.com/c/ymiB3v/N1Jf4) |
| Maize (*Zea mayz*) | Plant | Adaptation to highland | Ref. [31](https://paperpile.com/c/ymiB3v/DEHuS) |
| Groundsel (*Senecio lautus*) | Plant | Sand dune - Rocky highland ecotypes | Ref. [32](https://paperpile.com/c/ymiB3v/ifESA) |

**References**

1. Chung O., S. Jin, Y.S. Cho, *et al.* 2015. The first whole genome and transcriptome of the cinereous vulture reveals adaptation in the gastric and immune defense systems and possible convergent evolution between the Old and New World vultures. *Genome Biol.* **16**: 215.

2. Bassham S., J. Catchen, E. Lescak, *et al.* 2018. Repeated selection of alternatively adapted haplotypes creates sweeping genomic remodeling in stickleback. *Genetics* **209**: 921–939.

3. Jones F.C., Y.F. Chan, J. Schmutz, *et al.* 2012. A Genome-wide SNP genotyping array reveals patterns of global and repeated species-pair divergence in Sticklebacks. *Curr. Biol.* **22**: 83–90.

4. Stuart Y.E., T. Veen, J.N. Weber, *et al.* 2017. Contrasting effects of environment and genetics generate a continuum of parallel evolution. *Nat Ecol Evol* **1**: 158.

5. Deagle B.E., F.C. Jones, Y.F. Chan, *et al.* 2012. Population genomics of parallel phenotypic evolution in stickleback across stream-lake ecological transitions. *Proc. Biol. Sci.* **279**: 1277–1286.

6. Jeffery N.W., R.R.E. Stanley, B.F. Wringe, *et al.* 2017. Range-wide parallel climate-associated genomic clines in Atlantic salmon. *R Soc Open Sci* **4**: 171394.

7. Perrier C., V. Bourret, M.P. Kent, *et al.* 2013. Parallel and nonparallel genome-wide divergence among replicate population pairs of freshwater and anadromous Atlantic salmon. *Mol. Ecol.* **22**: 5577–5593.

8. Rougemont Q., P.-A. Gagnaire, C. Perrier, *et al.* 2017. Inferring the demographic history underlying parallel genomic divergence among pairs of parasitic and nonparasitic lamprey ecotypes. *Mol. Ecol.* **26**: 142–162.

9. Meier J.I., D.A. Marques, C.E. Wagner, *et al.* 2018. Genomics of parallel ecological speciation in Lake Victoria cichlids. *Mol. Biol. Evol.* **35**: 1489–1506.

10. Kautt A.F., K.R. Elmer & A. Meyer. 2012. Genomic signatures of divergent selection and speciation patterns in a “natural experiment”, the young parallel radiations of Nicaraguan crater lake cichlid fishes. *Mol. Ecol.* **21**: 4770–4786.

11. Reid N.M., D.A. Proestou, B.W. Clark, *et al.* 2016. The genomic landscape of rapid repeated evolutionary adaptation to toxic pollution in wild fish. *Science* **354**: 1305–1308.

12. Kozak G.M., R.S. Brennan, E.L. Berdan, *et al.* 2014. Functional and population genomic divergence within and between two species of killifish adapted to different osmotic niches. *Evolution* **68**: 63–80.

13. Lamichhaney S., A.P. Fuentes-Pardo, N. Rafati, *et al.* 2017. Parallel adaptive evolution of geographically distant herring populations on both sides of the North Atlantic Ocean. *Proc. Natl. Acad. Sci. U. S. A.* **114**: E3452–E3461.

14. Bradbury I.R., S. Hubert, B. Higgins, *et al.* 2010. Parallel adaptive evolution of Atlantic cod on both sides of the Atlantic Ocean in response to temperature. *Proc. Biol. Sci.* **277**: 3725–3734.

15. Pfenninger M., S. Patel, L. Arias-Rodriguez, *et al.* 2015. Unique evolutionary trajectories in repeated adaptation to hydrogen sulphide-toxic habitats of a neotropical fish (Poecilia mexicana). *Mol. Ecol.* **24**: 5446–5459.

16. Fraser B.A., A. Künstner, D.N. Reznick, *et al.* 2015. Population genomics of natural and experimental populations of guppies (*Poecilia reticulata*). *Mol. Ecol.* **24**: 389–408.

17. Zhao L. & D.J. Begun. 2017. Genomics of parallel adaptation at two timescales in Drosophila. *PLoS Genet.* **13**: e1007016.

18. Nadeau N.J., M. Ruiz, P. Salazar, *et al.* 2014. Population genomics of parallel hybrid zones in the mimetic butterflies, *H. melpomene* and *H. erato*. *Genome Res.* **24**: 1316–1333.

19. Yang Z., H. Zhong, J. Chen, *et al.* 2016. A Genetic mechanism for convergent skin lightening during recent human evolution. *Mol. Biol. Evol.* **33**: 1177–1187.

20. Foll M., O.E. Gaggiotti, J.T. Daub, *et al.* 2014. Widespread signals of convergent adaptation to high altitude in Asia and America. *Am. J. Hum. Genet.* **95**: 394–407.

21. Waterhouse M.D., L.P. Erb, E.A. Beever, *et al.* 2018. Adaptive population divergence and directional gene flow across steep elevational gradients in a climate-sensitive mammal. *Mol. Ecol.* **27**: 2512–2528.

22. Ravinet M., A. Westram, K. Johannesson, *et al.* 2016. Shared and nonshared genomic divergence in parallel ecotypes of Littorina saxatilis at a local scale. *Mol. Ecol.* **25**: 287–305.

23. Westram A.M., J. Galindo, M. Alm Rosenblad, *et al.* 2014. Do the same genes underlie parallel phenotypic divergence in different Littorina saxatilis populations? *Mol. Ecol.* **23**: 4603–4616.

24. Kess T., J. Galindo & E.G. Boulding. 2018. Genomic divergence between Spanish Littorina saxatilis ecotypes unravels limited admixture and extensive parallelism associated with population history. *Ecol. Evol.* **8**: 8311–8327.

25. Kubota S., T. Iwasaki, K. Hanada, *et al.* 2015. A genome scan for genes underlying microgeographic-scale local adaptation in a wild Arabidopsis species. *PLoS Genet.* **11**: e1005361.

26. Trucchi E., B. Frajman, T.H.A. Haverkamp, *et al.* 2017. Genomic analyses suggest parallel ecological divergence in Heliosperma pusillum (Caryophyllaceae). *New Phytol.* **216**: 267–278.

27. Huang Z., N.D. Young, M. Reagon, *et al.* 2017. All roads lead to weediness: Patterns of genomic divergence reveal extensive recurrent weedy rice origins from South Asian Oryza. *Mol. Ecol.* **26**: 3151–3167.

28. Fahrenkrog A.M., L.G. Neves, M.F.R. Resende Jr, *et al.* 2017. Population genomics of the eastern cottonwood (*Populus deltoides*). *Ecol. Evol.* **7**: 9426–9440.

29. Yeaman S., K.A. Hodgins, K.E. Lotterhos, *et al.* 2016. Convergent local adaptation to climate in distantly related conifers. *Science* **353**: 1431–1433.

30. Steane D.A., B.M. Potts, E.H. McLean, *et al.* 2017. Genomic scans across three Eucalypts suggest that adaptation to aridity is a genome-wide phenomenon. *Genome Biol. Evol.* **9**: 253–265.

31. Takuno S., P. Ralph, K. Swarts, *et al.* 2015. Independent molecular basis of convergent highland adaptation in Maize. *Genetics* **200**: 1297–1312.

32. Roda F., H. Liu, M.J. Wilkinson, *et al.* 2013. Convergence and divergence during the adaptation to similar environments by an australian groundsel. *Evolution* **67**: 2515–2529.
